# S100A4 makes two appearances in mechanisms leading to fibrosis

**DOI:** 10.1016/j.jbc.2024.107385

**Published:** 2024-05-15

**Authors:** László Nyitray

**Affiliations:** Department of Biochemistry, ELTE Eötvös Loránd University, Budapest, Hungary

## Abstract

Non-muscle myosin 2 (NM2) is known to play an important role in myofibroblast transdifferentiation, a hallmark of fibrotic disorders. In a recent JBC article, Southern *et al.* demonstrate that endogenous S100A4, a calcium- and NM2-binding protein acts as a mechanoeffector in this process. Since extracellular S100A4 is also involved in fibrogenesis by triggering the inflammatory response, this small protein appears to contribute to fibrosis *via* at least two distinct mechanisms.

Mechanosensing, which is the ability to respond to mechanical cues from the environment, is often key to the survival of cells. It plays important roles in both health and diseases such as organ fibrosis. Mechanotransduction dysregulation (alterations in signaling pathways from mechanical cues to cellular responses) after organ injuries often leads to fibroblast over-activation and differentiation to myofibroblasts (1). A better understanding of the key players involved in this process and the underlying mechanisms of fibrogenesis is of high interest for both basic and translational research.

The effector branch of mechanotransduction and transdifferentiation involves the extensive cytoskeletal remodeling of quiescent fibroblasts. Stress fibers appear and pre-myofibroblasts become motile. In stress fibers, α-smooth muscle actin replaces the β-actin isoform with a concomitant high contractile force and non-migratory phenotype (1). Non-muscle myosin 2 (NM2) proteins are the engine of these contractile bundles, and they are turned on and off by Ca- and calmodulin-dependent myosin light chain kinase (MLCK) and myosin light chain phosphatase, respectively.

The Olman research group has previously shown that NM2 proteins are directly involved as effector molecules in myofibroblast transdifferentiation. They have established a novel assay to measure fibroblast behavior in response to varying stiffness of the matrix and demonstrated that in normal lung tissue with organized and “soft” matrix substrate, cortical NM2 drives polarized migration. In contrast, in the stiff fibrotic matrix, mechanotransduction leads to diffusely activated stress fibers, increased intracellular tension, immobilized cells, and thus progressive myofibroblast formation (2).

In their recent JBC paper Southern and collaborators extend their work to reveal the critical role of S100A4, a small Ca-binding protein, as a key mechanoeffector in idiopathic pulmonary fibrosis (3). Since it is known that S100A4 binds one of the NM2 isoforms, NM2A, causing filament remodeling (4), the authors reasoned that this interaction could mediate the redistribution of the actomyosin complex leading to a pro-fibrotic phenotype.

The authors provide sufficiently compelling evidence to support their original assumption. They clearly demonstrate by loss- and gain-of-function experiments that S100A4 is upregulated at the pathophysiological range of extracellular matrix (ECM) stiffness and, through interactions with NM2A, triggers the redistribution of myosin filaments from the periphery to a central location. This Ca-dependent NM2A redistribution induces cellular stiffness, and thus myofibroblast differentiation. Only endogenous—and not extracellularly added—S100A4 regulates cell spreading in response to substrate stiffness. Their results also showed that S100A4 mediates the functional response to increasing substrate stiffness by growing focal adhesions and by increasing cell contractility. Furthermore, S100A4 KO mice are protected from the pro-fibrotic effects of bleomycin.

The researchers provide solid experimental evidence of the key role of intracellular S100A4 in fibrogenesis *via* its isoform-specific interaction with NM2A. S100A4 binding to the C-terminal end of the coiled coil tail and part of the disordered “tailpiece” of the NM2A heavy chain leads to filament disassembly (5) at the cortical region, where the local Ca-concentration is increased by opening of mechanosensitive Ca-channels (1). It is worth noting that the cortical NM2 filaments in quiescent fibroblasts are heterotypic (6), *that is*, contain a mixture of NM2A and NM2B isoforms. Interestingly, NM2B distribution remains constant during transdifferentiation, consistent with the previously suggested mutually exclusive regulation of the two isoforms: S100A4 binding and heavy chain phosphorylation regulates filament disassembly in NM2A and NM2B, respectively (7). The disassembled, inactive and folded NM2A monomers bound to S100A4 could diffuse to the central region of the cell, where the decrease in Ca-concentration allows dissociation of S100A4 and reassembly of NM2A-containing filaments, ultimately leading to myofibroblast-specific actomyosin stress fibers (Fig. 1).

**Figure 1:**
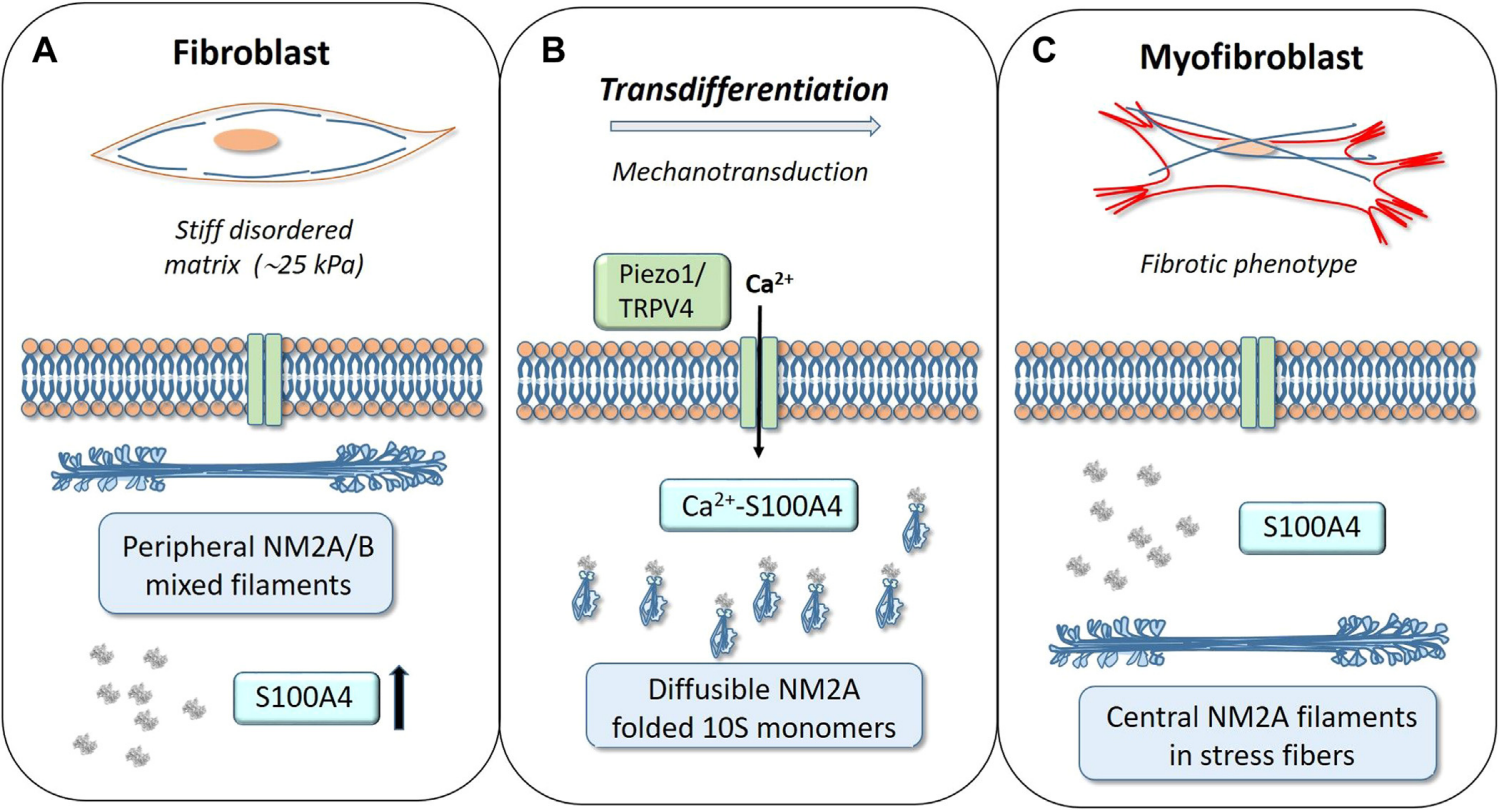
**Schematic view of the role of intracellular S100A4 in myofibroblast transdifferentiation.***A*, pulmonary fibroblasts in pathophysiological ECM stiffness overexpress S100A4. Mixed NM2 filaments are part of the cortical cytoskeleton. *B*, mechanosensitive Ca-channels (Piezo1/TRPV4) open and the increase in local Ca-concentration activates S100A4. S100A4 binds to NM2A and disrupts filaments, allowing the monomeric 10S NM2A to diffuse to the cell center. *C*, as local Ca-concentration drops, NM2A filaments reassemble to function in stress fibers of the fully differentiated myofibroblasts.

The most intriguing conclusion based on these findings (3) relates to the role of S100A4 in fibrogenesis. S100 proteins are an enigmatic, evolutionarily young, vertebrate-specific protein family with more than 20 paralogs. What could be the function of so many Ca-binding proteins in vertebrates? In fact, we know more about their pathological rather than their physiological roles. Their overexpression is associated with many diseases including cancers, chronic inflammatory disorders, and organ fibrosis (8). What do we know about the physiological role of S100A4? It facilitates migration by modulating NM2A turnover at the leading edge in various cell types (3, 4) and reduces tissue surface tension in 3D spheroid cell cultures (9).

To make the picture even more complicated, S100 proteins, including S100A4, are released from cells by a mostly unknown mechanism(s) with a variety of extracellular roles. S100A4 (together with other isoforms) is considered a damage-associated molecular patterns protein that alerts the surrounding tissue of danger by interacting with pattern recognition receptors such as Toll-like receptor 4 and receptor for advanced glycation end product. These interactions in turn trigger multiple downstream pathways involved in inflammation and fibrosis. Several publications provide experimental evidence that extracellular S100A4 is intimately involved in various types of organ fibrosis ((10) and references therein). Released S100A4 could trigger neighboring fibroblasts to differentiate into myofibroblasts and stimulate neighboring cells to produce and secrete more S100A4 in a feed-forward manner.

It remains to be seen in future studies whether these two, apparently completely different mechanisms have synergistic or independent roles in myofibroblast transdifferentiation and/or other aspects of fibrotic disorders. Both extra- and intracellular S100A4 seem to be key players in fibrotic disorders and both are potentially attractive targets for therapeutic intervention. Neutralizing antibodies against extracellular S100A4 are in clinical trials (10), and S100A4-specific small molecule inhibitors are also under development. Finally, it should be taken into account when trying to unravel the role of S100 proteins in physiological processes and as a target for therapeutic purposes, that other family members are also expressed in fibroblasts, and since S100 possesses some functional redundancy (7), their possible contribution to myofibroblast transdifferentiation needs to be explored.

## Conflict of interest

The author declares that they have no conflicts of interest with the contents of this article.
